# Prothrombotic state in senile patients with acute exacerbations of chronic obstructive pulmonary disease combined with respiratory failure

**DOI:** 10.3892/etm.2013.919

**Published:** 2013-01-22

**Authors:** YA-JUN SONG, ZHE-HUI ZHOU, YAO-KANG LIU, SHI-MING RAO, YING-JUN HUANG

**Affiliations:** Department of Respiratory Medicine, Huangpu District Central Hospital, Shanghai 200002, P.R. China

**Keywords:** acute exacerbations of chronic obstructive pulmonary disease, respiratory failure, fibrinogen, D-dimer, hemorheology, low molecular weight heparin

## Abstract

The aim of this study was to study the clinical value of prethrombotic state and treatment with low molecular weight heparin (LMWH) in senile patients with acute exacerbations of chronic obstructive pulmonary disease (AECOPD) combined with respiratory failure. Hemorheological markers (hematocrit, blood viscosity and plasma viscosity), fibrinogen (FIB), D-dimer and gas analysis were evaluated in 30 senile patients with AECOPD combined with respiratory failure and compared with those in 30 cases without respiratory failure. A total of 30 cases with AECOPD combined with respiratory failure were randomly divided into treatment and control groups. The two groups received conventional treatment. The treatment group also received LMWH injections every 12 h for 6 days and the clinical effect was observed. The levels of FIB, D-dimer, hematocrit, blood viscosity and plasma viscosity were significantly higher in the patients with AECOPD combined with respiratory failure compared with those in the patients without respiratory failure. The plasma D-dimer and FIB levels had significantly positive correlations with the partial pressure of CO_2_ (PaCO_2_) and negative correlations with the partial pressure of O_2_ (PaO_2_) in the patients with AECOPD combined with respiratory failure. The curative effect was improved in the treatment group, compared with that in the control group without side-effects. However, no significant changes in activated partial thromboplastin time (APTT) and international normalized ratio (INR) were observed between the treatment and control groups. The senile patients with AECOPD combined with respiratory failure suffered from hypercoagulation. Early detection and diagnosis of the prethrombotic state and timely treatment with LMWH may benefit these patients without side-effects.

## Introduction

Although chronic obstructive pulmonary disease (COPD) is not a high risk factor for pulmonary embolism (PE), acute exacerbations of chronic obstructive pulmonary disease (AECOPD) complicated with PE occur in numerous patients. The correlation between AECOPD and PE has been a key research topic. The pathophysiological changes in COPD are as follows: i) reconstruction of the pulmonary vascular bed: extensive contraction and arterial hypertension caused by long-term hypoxia, vascular intimal hyperplasia and vascular fibrosis and occlusion; and ii) blood hypercoagulability: blood stasis caused by increased erythrocyte formation due to long-term hypoxia, vascular endothelial cell dysfunction, pulmonary heart disease combined with right ventricular dysfunction, dependence on corticosteroids and increased concentrations of blood-clotting substances. These changes cause venous blood stasis, vein endothelial injury and blood hypercoagulability (the three elements of Virchow’s triad), leading to formation of the prethrombotic state (PTS).

AECOPD is often complicated with respiratory failure. In the majority of patients, due to hypoxemia and carbon dioxide retention (1–3), the blood is in a hypercoagulable or PTS state, with the formation of small pulmonary artery thrombi leading to a poor prognosis (4–6). For elderly patients the less stable internal environment, decreased defense against external infection and organ aging cause a higher incidence of AECOPD. The change in the illness condition is characterized by acute onset, severe condition and poor drug reaction. Consequently respiratory failure occurs more easily. It has been observed that the incidences of pulmonary infarction and disseminated intravascular coagulation (DIC) are higher in elderly patients with AECOPD complicated with respiratory failure, and this has been a significant cause of increased mortality (7–9). There are multiple risk factors, such as senility, being bedridden and vascular injury induced by interventional surgery during hospitalization, which frequently lead to the PTS state. Therefore, intervention in thrombus formation is essential.

At present, research on the PTS state in elderly patients with AECOPD complicated with respiratory failure is seldom reported. In the present study, simple detection methods were used to observe blood hypercoagulability and hyperviscosity and assess the severity of illness. The incidence of PE was reduced by timely anticoagulation therapy. The objective of this study was to identify an appropriate and effective treatment for elderly patients with AECOPD complicated with respiratory failure, thus reducing the mortality and improving the physical and mental health and quality of life of patients.

## Patients and methods

### Subjects

A total of 60 elderly patients with AECOPD were admitted and treated at the Huangpu District Central Hospital between January 2010 and December 2011. The present study was conducted in accordance with the Declaration of Helsinki and with approval from the Ethics Committee of Huangpu District Central Hospital. Written informed consent was obtained from all participants. The patients were treated at the respiratory and geriatric special procurement departments of the hospital. Patients were evaluated according to medical history, physical examination and various physical and laboratory analyses and divided into AECOPD with type II respiratory failure and AECOPD without type II respiratory failure groups. There were 30 patients in each group. AECOPD diagnosis was in line with China’s 2007 edition of the COPD treatment guidelines (9); diagnosis of type II respiratory failure was in accordance with diagnosis standards: in a patient at rest, breathing air at sea level, a partial pressure of O_2_ (PaO_2_) <60 mmHg and a partial pressure of CO_2_ (PaCO_2_) >50 mmHg. In the AECOPD with type II respiratory failure group, there were 21 males and 9 females, aged between 74 and 85 years, with an average age of 79.27±3.43 years. In the AECOPD without type II respiratory failure group, there were 20 males and 10 females, aged between 74 and 85 years, with an average age of 80.17±3.04 years. The differences of age and gender between the two groups were not statistically significant (P>0.05).

The 30 patients in the AECOPD with type II respiratory failure group were randomly divided into a low molecular weight heparin (LMWH) treatment and conventional treatment group. There were 15 patients in each group. In the LMWH treatment group, there were 11 males and 4 females, aged between 75 and 85 years, with an average age of 79.27±3.43 years. In the conventional treatment group, there were 10 males and 5 females, aged between 74 and 85 years, with an average age of 79.80±3.78 years. The differences of age and gender between the two groups were not statistically significant (P>0.05).

All patients with blood coagulation dysfunction caused by conditions such as liver and blood diseases and splenomegaly were excluded. Drugs which affect blood coagulation function were not used during the two weeks of blood collection.

### Methods

For all patients, venous and arterial blood was extracted on an empty stomach early in the morning. Hematocrit, whole blood viscosity (high and low shear), plasma viscosity, fibrinogen (FIB), D-dimer, activated partial thromboplastin time (APTT), international normalized ratio (INR) and blood gas analysis were then evaluated. Patients in the AECOPD with type II respiratory failure group were randomly divided into a LMWH treatment and conventional treatment group. On the 7th day following treatment, blood collection was performed again and the parameters of the previously mentioned indicators were measured.

Hematocrit was tested by the capillary method and the detection instrument was an EHK-40 sedimentation hematocrit instrument; whole blood viscosity and plasma viscosity were tested by the rotary viscometer method and the detection instrument was a Succeeder-SA-6000; FIB, APTT and INR were tested by the solidification method and the detection instrument was a Sysmex CA-510 coagulation analyzer (Kobe, Japan); D-dimer was tested by the colloidal gold assay method and the detection instrument was an Uppergold U2 reader; arterial blood gas was analyzed by the electrode method and the detection instrument was an ABL80 blood gas analyzer (Radiometer, Brønshøj, Denmark).

All patients in the LMWH and conventional treatment groups were treated with conventional treatment. The conventional treatment included continuous low-flow oxygen, anti-infectives, bronchial antispasmodics, expectorants, respiratory stimulants, correctors of acid-base and electrolyte imbalance and the reasonable application of glucocorticoids, cardiotonics, diuretics, vasodilators and other symptomatic supportive treatments. Other drugs that affected coagulation function were not included. Patients in the LMWH treatment group received LMWH calcium treatment in addition to conventional treatment. LMHW was subcutaneously injected at a dose of 4,000 Units, twice a day, for 6 days. Blood samples were collected before LMWH treatment and on the 7th day after treatment and the previously mentioned indicators were subsequently measured. Patients in the conventional treatment group received only conventional treatment, without LMWH treatment. The detection methods and measurement times of the associated indicators were the same as those of the LMWH treatment group.

### Statistical analysis

The measurement data were expressed as the mean ± SD. The data which were in line with the normal distribution were tested by t-tests, while the data which were in line with a skewed distribution were tested by the Wilcoxon test. Comparisons between the AECOPD with type II respiratory failure and AECOPD without type II respiratory failure groups were tested by t-tests, independent sample tests and NPar tests. Nonparametric correlations were used for the analyses of correlations between D-dimer, FIB, PaCO_2_ and PaO_2_ in the AECOPD with type II respiratory failure group. Comparisons between data before and after treatment were tested by paired sample tests and Wilcoxon signed ranks tests. Comparisons of curative effect between the LMWH and conventional treatment groups were tested by t-tests, independent sample tests, NPar tests and Mann-Whitney tests. P<0.05 was considered to indicate a statistically significant difference.

## Results

### General data

Compared with the AECOPD without respiratory failure group, the AECOPD with respiratory failure group had higher hematocrit, plasma viscosity, whole blood viscosity (including high and low shear), FIB and D-dimer levels. The differences were significant (P<0.01; Table I).

### Correlation analyses

For patients in the AECOPD with respiratory failure group (30 cases), correlations of D-dimer and FIB with PaCO_2_ and PaO_2_ were observed in the present study. The results showed that D-dimer and FIB levels were positively correlated with PaCO_2_ and negatively correlated with PaO_2_ (Table II).

### Before and after treatment

The conditions of the patients in the LMWH and conventional treatment groups were noticeably improved following treatment. Compared with the conventional treatment group, the hematocrit, whole blood viscosity (high shear), whole blood viscosity (low shear), plasma viscosity, FIB, PaO_2_ and PaCO_2_ of the LMWH treatment group were clearly improved. The differences were significant (P<0.05). The D-dimer levels of the patients in the two groups were decreased following therapy. Compared with the conventional treatment group, the LMWH group was improved noticeably, but the difference between groups was not significant (P>0.05). APTT and INR lengthened within the acceptable range and the difference between the groups was not significant (P>0.05). No serious bleeding events occurred (Table III). However, in the conventional treatment group, one patient was complicated by cerebral infarction and another by lower limb vein embolism.

## Discussion

Elderly patients with COPD often suffer from complications due to underlying diseases, such as hypertension, coronary heart disease, cardiac insufficiency, diabetes and cerebral infarction. These diseases cause hypercoagulability and viscosity of the blood. When elderly patients with AECOPD are complicated with respiratory failure, hypercoagulability of the blood occurs and minute thrombi form due to various causes. There are often risk factors which cause venous thromboembolism, such as being bedridden in the long-term, repeated bronchopulmonary infection, right heart failure and vein drainage disturbance. These make the condition more complex, increase complications and lead to a worse prognosis. Further complications may arise during the hospitalization process due to the acute exacerbation period, vessel damage caused by deep vein puncture and the use of diuretics which also increase the risk of phlebothrombosis.

For certain thrombosis-related diseases, a pathological process called the PTS state often exists in the blood prior to thrombosis, which refers to the visible pathological changes in the biochemistry and rheology of the blood or intangible components caused by a variety of factors (1,2). COPD has the three key factors for phlebothrombosis formation, vascular endothelial injury, increased blood viscosity and blood stasis, and it is a type of PTS disease (6,10–12). Preliminary studies have been performed by the present research group and it was demonstrated that elderly patients with COPD had a certain degree of hypercoagulability and viscosity in the relatively stable period as well as the acute exacerbation period, which was directly associated with repeated infections, hypoxia and hypercapnia.

The hyperviscosity state of patients with AECOPD complicated by respiratory failure was more serious than that of patients without respiratory failure. It was demonstrated in the present study that the hematocrit, plasma viscosity and whole blood viscosity (including high and low shear) of elderly patients with AECOPD complicated by respiratory failure increased. Compared with the AECOPD without respiratory failure group, the differences were significant. In blood viscosity measurements, it is generally acknowledged that high-shear-specific viscosity represents red cell deformability and low-shear-specific viscosity mainly reflects the aggregation of red cells. Patients with AECOPD, particularly those complicated by respiratory failure, are usually elderly. The decline in lung function and respiratory failure may limit activity and lead to the patient being bedridden in the long-term. Furthermore, hypoxia, malnutrition and other COPD-inherent pathophysiological phenomena also increase blood viscosity.

The hypercoagulability of patients with AECOPD complicated by respiratory failure was more serious compared with that of the patients without respiratory failure; thromboses may be present. It was indicated in the present study that the levels of D-dimer and FIB in the elderly patients of the AECOPD complicated by respiratory failure group were noticeably higher than those of the AECOPD without respiratory failure group. FIB is a glycoprotein synthesized by the liver. Agnelli *et al* (13) indicated that an increased FIB level was an important risk factor for thrombosis and cardiovascular diseases. D-dimer is the specific degradation product of cross-linked fibrin and is used as a molecular marker of hypercoagulation and secondary increased fibrinolytic activity (14–16). In addition, it was shown in the present study that the D-dimer and FIB levels of the respiratory failure group were positively correlated with PaCO_2_ and negatively correlated with PaO_2_. This indicated that the rise in D-dimer and FIB levels in the plasma of patients with respiratory failure had a close correlation with the degree of hypoxemia and hypercapnia and reflected the severity of the condition.

As mentioned previously, PTS gradually occurred in the pathophysiological process of COPD and there was a vicious cycle between PTS and COPD. Due to the existence of PTS, COPD was not only a simple airway or lung disease but also a vascular disease, particularly in elderly patients with AECOPD complicated by respiratory failure. PTS increased the risk of deep vein thrombosis and pulmonary thromboembolism and promoted the occurrence and development of pulmonary hypertension and pulmonary heart disease. Furthermore, PTS increased the occurrence of cardiovascular and cerebrovascular events in patients with COPD. In the present study, there were two elderly patients with AECOPD complicated by respiratory failure who were also complicated by pulmonary heart disease. Within two weeks after treatment, one patient was complicated by cerebral infarction and the other was complicated by lower limb vein embolism, which also indicated the high occurrence of vascular diseases in elderly patients with AECOPD complicated by respiratory failure.

It has been widely demonstrated that hyperviscosity and hypercoagulability occur in the blood of patients with AECOPD (17,18), which provided a theoretical basis for the clinical detection of PTS in patients with AECOPD and the rational use of anticoagulants. PTS in elderly patients with AECOPD complicated by respiratory failure is more serious. Therefore, for elderly patients with AECOPD complicated by respiratory failure, the timely use of anticoagulation treatments at the time of conventional treatment may improve the ventilation/perfusion ratio and the hypoxic state of the patient and prevent cardiovascular and cerebrovascular events.

LMWH is a novel antithrombin III-dependent anti-thrombosis drug. It is a heparin fragment generated via chemical or enzymatic depolymerization. Its size is ∼one-third that of the unfractionated heparin. The T_1/2_ of LMWH is ∼8-fold of that of unfractionated heparin (19). Therefore, LMWH has the following advantages for anticoagulation therapy: i) it may be absorbed easily via subcutaneous injection; ii) it has a specific anticoagulant effect; and iii) hemorrhage side-effects are reduced and monitoring of coagulation function is not required. It was shown in the present study that a number of indicators of patients in the LMWH treatment group were improved more clearly following treatment. Compared with the conventional treatment group, hematocrit, whole blood viscosity (high shear), whole blood viscosity (low shear), plasma viscosity, FIB, PaO_2_ and PaCO_2_ were improved noticeably and the differences were significant. This indicated that anticoagulant therapy has a certain effect on PTS improvement in elderly patients with AECOPD complicated by respiratory failure. It was also revealed in the present study that the D-dimer levels of patients in the two groups decreased after treatment. The LMWH group was improved more markedly than the conventional treatment group, although the differences between the groups were not significant. In addition, the safety of LMWH anticoagulation was monitored in the present study and it was observed that APTT and INR lengthened within the acceptable range. The differences between the groups were not significant. No serious bleeding events occurred, indicating the effectiveness and safety of the LMWH treatment.

During the present study, there were two elderly patients with AECOPD complicated by respiratory failure and pulmonary heart disease in the conventional treatment group. Of the two patients, one was complicated by cerebral infarction and the other by lower limb vein embolism within two weeks after treatment, while there were no such complications in the LMWH treatment group. This suggested that anticoagulation intervention therapy for elderly patients with AECOPD complicated by respiratory failure in the early stage may be effective in preventing the occurrence of vascular diseases.

In conclusion, PTS existed in elderly patients with AECOPD complicated by respiratory failure. The monitoring of plasma D-dimer, FIB and hemorheology was simple and aided in understanding the hyperviscosity, hypercoagulability and fibrinolytic state of the elderly patients with AECOPD complicated by respiratory failure, which provided an objective basis for early interventions. The prophylactic use of LMWH in the early stage had certain clinical significance for the remission of patient PTS, decrease of vascular disease incidence and improvement of patient prognoses.

## Figures and Tables

**Table I:** Comparisons of hemorheology, FIB, D-dimer and blood gas analysis between the AECOPD with respiratory failure and AECOPD without respiratory failure groups.

Parameter	With respiratory failure (n=30)	Without respiratory failure (n=30)
Hematocrit (%)	45.97±7.79	38.25±6.44^*^
Plasma viscosity (mPas)	1.57±0.22	1.34±0.23^*^
Whole blood viscosity (high shear; 200 s^−1^)	4.56±0.58	4.13±0.59^*^
Whole blood viscosity (low shear; 1 s^−1^)	18.72±2.88	16.38±2.19^*^
FIB (g/l)	4.40±0.64	3.20±0.64^*^
D-dimer (mg/l)	0.36±0.26	0.11±0.08^*^
PaO_2_ (mmHg)	52.53±8.01	79.73±10.77^*^
PaCO_2_ (mmHg)	85.27±16.08	45.07±8.58^*^

Comparisons were between the COPD with respiratory failure and COPD without respiratory failure groups:

* P<0.01. AECOPD, acute exacerbations of chronic obstructive pulmonary disease; FIB, fibrinogen, PaO_2_, partial pressure of O_2_; PaCO_2_, partial pressure of CO_2_.

**Table II:** Analyses of the correlations of FIB and D-dimer with PaCO_2_ and PaO_2_ in the AECOPD with respiratory failure group.

Comparison	r	P-value
FIB and PaO_2_	−0.73	<0.01
FIB and PaCO_2_	0.66	<0.01
D-dimer and PaO_2_	−0.80	<0.01
D-dimer and PaCO_2_	0.62	<0.01

AECOPD, acute exacerbations of chronic obstructive pulmonary disease; FIB, fibrinogen, PaO_2_, partial pressure of O_2_; PaCO_2_, partial pressure of CO_2_.

**Table III:** Comparisons of the parameters of the low molecular weight heparin and conventional treatment groups before and after treatment.

Factor	Low molecular weight heparin treatment group (n=15)		Conventional treatment group (n=15)	
	
	Before treatment	After treatment	Before treatment	After treatment
Hematocrit (%)	45.39±8.37	36.88±5.00^*Δ^	46.56±7.41	44.04±7.68^*^
Plasma viscosity (mPas)	1.58±0.23	1.36±0.13^*Δ^	1.55±0.22	1.52±0.15
Whole blood viscosity (high shear; 200 s^−1^)	4.60±0.58	3.60±0.40^*Δ^	4.51±0.59	4.21±0.31^*^
Whole blood viscosity (low shear; 1 s^−1^)	18.68±2.82	16.19±1.46^*Δ^	18.76±3.04	17.93±1.70
FIB (g/l)	4.45±0.64	3.20±0.48^*Δ^	4.36±0.66	4.11±0.44^*^
D-dimer (mg/l)	0.37±0.25	0.07±0.05^*^	0.36±0.28	0.17±0.06^*^
PaO_2_ (mmHg)	52.53±8.31	83.73±7.92^*Δ^	52.53±7.98	74.40±8.72^*^
PaCO_2_ (mmHg)	86.00±17.01	46.07±6.31^*Δ^	84.53±15.66	57.07±9.48^*^
APTT (S)	34.02±6.31	40.04±6.88^*^	32.98±5.67	38.98±6.55^*^
INR	0.98±0.14	1.08±0.18^*^	1.00±0.13	1.14±0.22^*^

* P<0.05, compared with data before treatment,

Δ P<0.05, compared with the control group. FIB, fibrinogen, PaO_2_, partial pressure of O_2_; PaCO_2_, partial pressure of CO_2_; APTT, activated partial thromboplastin time; INR, international normalized ratio.

## References

[1] Macnee W (2007). Pathogenesis of chronic obstructive pulmonary disease. Clin Chest Med.

[2] Rizkallah J, Man SF, Sin DD (2009). Prevalence of pulmonary embolism in acute exacerbations of COPD: a systematic review and metaanalysis. Chest.

[3] Ashitani J, Mukae H, Arimura Y, Matsukura S (2002). Elevated plasma procoagulant and fibrinolytic markers in patients with chronic obstructive pulmonary disease. Intern Med.

[4] Lim W, Eikelboom JW, Ginsberg JS (2007). Inherited thrombophilia and pregnancy associated venous thromboemlism. BMJ.

[5] Dahl M, Tybjaerg Hansen A, Vestbo J, Lange P, Nordestgaard BG (2001). Elevated plasma fibrinogen associated with reduced pulmonary function and increased risk of chronic obstructive pulmonary disease. Am J Respir Crit Care Med.

[6] Blom JW, Doggen CJ, Osanto S, Rosendaal FR (2005). Maligancies, prothrombotic mutations, and the risk of venous thrombosis. JAMA.

[7] Torbicki A, Perrier A, Konstantinides S (2008). Guidelines on the diagnosis and management of acute pulmonary embolism: the Task Force for the Diagnosis and Management of Acute Pulmonary Embolism of the European Society of Cardiology (ESC). Eur Heart J.

[8] Sin DD, Man PSF (2003). Why are patients with chronic obstructive pulmonary disease at increased risk of cardiovascular diseases? The potential role of systemic inflammation in chronic obstructive pulmonary disease. Circulation.

[9] GOLD Executive Committee Global strategy for the diagnosis, management, and prevention of chronic obstructive pulmonary disease (Revised 2011). http://www.goldcopd.org/.

[10] Wedzicha JA (2000). The heterogeneity of chronic obstructive pulmonary disease. Thorax.

[11] Gunen H, Gulbas G, In E, Yetkin O, Hacievliyagil SS (2010). Venous thromboemboli and exacerbations of COPD. Eur Respir J.

[12] Ambrosetti M, Ageno W, Spanevello A, Salerno M, Pedretti RF (2003). Prevalence and prevention of venous thromboembolism in patients with acute exacerbations of COPD. Thromb Res.

[13] Agnelli G, Verso M, Ageno W (2008). The MASTER registry on venous thromboembolism: description of the study cohort. Thromb Res.

[14] Mispelaere D, Glerant JC, Audebert M (2002). Pulmonary embolism and sibilant types of chronic obstructive pulmonary disease decompensations. Rev Mal Respir.

[15] Hartmann IJ, Hagen PJ, Melissant CF, Postmus PE, Prins MH (2000). Diagnosing acute pulmonary embolism: effect of chronic obstructive pulmonary disease on the performance of D-dimer testing, ventilation/perfusion scintigraphy, spiral computed tomographic angiography, and conventional angiography. ANTELOPE Study Group Advances in New Technologies Evaluating the Localization of Pulmonary Embolism. Am J Respir Crit Care Med.

[16] Shitrit D, Bendayan D, Bar-Gil-Shitrit A (2002). Significance of a plasma D-dimer test in patients with pulmonary hypertension. Chest.

[17] Singer DE, Albers Gw, Dalen JE (2008). Antithrombotic therapy in atrial fibrillation: American College of Chest Physicians Evidence-Based Clinical Practice Guidelines (8th Edition). Chest.

[18] Segal JB, Streiff MB, Hofmann LV, Thornton K, Bass EB (2007). Management of venous thromboembolism: a systematic review for a practice guideline. Ann Intern Med.

[19] Kleber FX, Witt C, Vogel G (2003). Randomized comparison of enoxaparin with unfractionated heparin for the prevention of venous thromboembolism in medical patients with heart failure or severe respiratory disease. Am Heart J.

